# Unmet needs of young adults following first-episode psychosis in KwaZulu-Natal, South Africa: Baseline findings from a pilot randomised controlled trial of basic income support

**DOI:** 10.4102/sajpsychiatry.v32i0.2455

**Published:** 2026-02-16

**Authors:** Joyce P. Mlay, Vuyokazi Ntlantsana, Neliswa Gcabashe, Lise Jamieson, Thirusha Naidu, Busisiwe S. Bhengu, Lindokuhle T. Thela, Saeeda Paruk, Jonathan K. Burns, Bonginkosi Chiliza, Richard Lessells, Andrew Tomita

**Affiliations:** 1 Discipline of Public Health Medicine, School of Medicine, University of KwaZulu-Natal, Durban, South Africa; 2 Health Economics and HIV and AIDS Research Division, University of KwaZulu-Natal, Durban, South Africa; 3 Discipline of Psychiatry, School of Medicine, University of KwaZulu-Natal, Durban, South Africa; 4 Discipline of Nursing, School of Health Sciences, University of KwaZulu-Natal, Durban, South Africa; 5 Health Economics and Epidemiology Research Office, School of Clinical Medicine, Faculty of Health Sciences, University of the Witwatersrand, Johannesburg, South Africa; 6 South African Centre for Epidemiological Modelling and Analysis (SACEMA), Stellenbosch University, Stellenbosch, South Africa; 7 Department of Innovation in Medical Education, Faculty of Medicine, University of Ottawa, Ottawa, Canada; 8 Department of Public Health and Primary Care, University of Cambridge, Cambridge, United Kingdom; 9 Faculty of Health and Life Sciences, University of Exeter, Exeter, United Kingdom; 10 Centre for the AIDS Programme of Research in South Africa, College of Health Sciences, University of KwaZulu-Natal, Durban, South Africa; 11 KwaZulu-Natal Research Innovation and Sequencing Platform (KRISP), College of Health Sciences, University of KwaZulu-Natal, Durban, South Africa; 12 Centre for Rural Health, School of Medicine, University of KwaZulu-Natal, Durban, South Africa

**Keywords:** FEP, unmet needs, young adults, universal basic income, pilot

## Abstract

**Background:**

People with psychosis have multiple and complex needs. The first episode of psychosis (FEP), as a distinct health challenge that occurs frequently during adolescence or early adult years, is a serious threat because of high levels of poverty among the youth in South Africa.

**Aim:**

This study quantifies the needs among unemployed FEP adults aged 18–29 years in South Africa for potential early intervention targeting.

**Setting:**

The study was conducted at government psychiatric facilities in Msunduzi Municipality, KwaZulu-Natal province, South Africa.

**Methods:**

As part of a pilot randomised controlled trial of an unconditional cash transfer (UCT) intervention, also known as basic income support (BIS), 60 FEP participants were enrolled, and we assessed their needs using the Camberwell Assessment of Needs, the Household Food Insecurity Access Scale and the Water Insecurity Experience Scale. Descriptive cross-sectional analyses were summarised for various domains of needs.

**Results:**

Most participants were black people (*n* = 58, 96.7%), male (*n* = 47, 78.3%), with a median age of 23 years (interquartile range [IQR] = 20.0–25.5), with half being diagnosed with schizophrenia (*n* = 30, 50%). The most significant severe unmet need was the inability to access government benefits to which one is entitled. Despite the limited availability of mental health services in South Africa, the majority reported that their need for managing psychotic symptoms was met or partially met.

**Conclusion:**

The needs of youth must go beyond the temporary management of psychotic symptoms.

**Contribution:**

This study highlights the unmet needs of young adults with FEP in resource-constrained environments and underscores the need for integrated interventions.

## Introduction

Psychosis is a debilitating health condition that often occurs in the early 20s and disrupts various aspects of life with long-term health consequences. The aetiology of psychosis is complex and multifaceted; although the biological and genetic causes of psychosis are inevitable, social, economic and cultural factors play a role in both causing and influencing the outcomes of the disease.^1,2^ In the mental health research debate, persistent health consequences of psychosis include relapse, treatment resistance, cognitive impairment, reduced social and occupational functioning, poor quality of life and stigma and discrimination.^3,4^ Individuals living with psychosis have a wide range of needs defined as ‘a requirement of an individual to achieve, maintain, or restore an acceptable level of social independence’.^5,6,7^ Exploring the type and extent of patients’ needs is a crucial component of patient-centred care, as it provides the basis for developing appropriate interventions, ensuring holistic support or treatment and improving overall long-term well-being.^5,8^

According to the social causation theory, income poverty increases the risk of developing mental disorders such as anxiety and depression.^9^ Conversely, the social drift theory posits that mental illness contributes to poverty, as affected individuals often experience reduced productivity and income, thereby deepening their economic disadvantage.^9^ Young people with first episode of psychosis (FEP) often face significant challenges, including being unskilled, unemployed and socially isolated. Although a few may have skills and employment, psychosis frequently leads to job loss.^10^ The effects of psychosis disrupt various aspects of life, creating a wide range of needs. The needs include medical and psychiatric care and social needs.^11^ To meet these needs and re-establish themselves, support may be required from family, friends and mental health services.^12^

For the youth in South Africa who are often unemployed or unskilled because of the nature of their young age,^13^ experiencing psychosis (for the first time) further compounds needs and future aspirations at a critical juncture when the transition from education to employment needs to take place for one to become a productive member of society. In South Africa (SA), 53.4% of youth aged 18–35 years living in rural areas are unemployed because of limited work opportunities.^14^ Although there is no estimate in (rural) South Africa, the unemployment among patients with FEP is 65% in the United States, even in a country with a traditionally high employment rate.^15^ Addressing poverty-related challenges requires financial support, with cash transfer interventions being one such approach. These interventions effectively encourage service utilisation and reduce structural barriers created by poverty, thereby supporting better health outcomes, including mental health. Cash grants are typically offered in two forms: conditional cash transfers (CCTs) and unconditional cash transfers (UCTs).^16,17^

Given complex and wide-ranging needs in a region with a high scarcity of community-based mental health services, support may be required from various sources, including family, friends and local non-mental health organisations (i.e. social services).^11,12^ Early interventions during the first signs/stages of FEP can be critical in reducing the morbidity associated with the illness and assisting with lowering rehospitalisation rates, improving symptom management and enhancing functioning and quality of life for those affected by psychosis.^18^ Minimising relapse is crucial as each episode is associated with cognitive changes and an increased risk of complications. To date, several studies have assessed the needs of individuals with severe mental illness (SMI) in the Western Cape and KwaZulu-Natal, South Africa, using the Camberwell Assessment of Needs. Still, neither study quantified the needs of FEP individuals.^8,19^ This study aims to determine young patients’ needs following the FEP and inform clinical practice by underscoring the importance of holistic care to enhance patient engagement and reduce the risk of relapse.

## Research methods and design

### Study setting

As part of a randomised controlled trial (RCT), named Poverty Reduction Strategy for First Episode Psychosis (PRS-FEP) trial^15^ (trial #DOH- 27- 092022- 5894), this current (baseline) assessment examined the needs of young patients following FEP and was conducted between July 2023 and December 2023 at government psychiatric facilities in Msunduzi Municipality, KwaZulu-Natal, South Africa. The hospitals are located in a municipality with a population of 679 000 people, 80% of whom are black Africans, over half live in rural areas and the unemployment rate is more than one in three people.^20^ Potential study participants were approached from the PSYchosis MAPing in KwaZulu-Natal (PSYMAP-ZN) cohort,^21^ with the PRS-FEP inclusion criteria being (1) positive FEP status, diagnosed by a psychiatrist under the ICD-11 criteria for psychotic disorders, (2) in remission, (3) on anti-psychotic medication for treatment for less than 6 months after initial psychiatric hospital discharge, (4) aged 18–29 years, (5) speaking isiZulu (language spoken in the study area) or English, (6) unemployed and (7) resident in the Msunduzi area for ≥ 6 months to ensure we engage only residents of Msunduzi and restrict those who only use the hospital service. First-episode psychosis is defined as the first presentation of psychosis meeting DSM 5 criteria for a psychotic disorder and the use of anti-psychotic medication for less than 6 months after the initial psychiatric visit.^22,23^ Remission is defined as the absence of positive and negative symptoms at the enrolment time or with mild symptoms (two or less positive or negative symptoms), as assessed by the PRS-FEP research assistant using the Positive and Negative Syndrome Scale (PANSS).^24^ The study exclusion criteria were a lack of written consent and capacity to participate in our study. A trained research assistant with an honours degree in psychology, fluent in isiZulu and English, provided individuals meeting these criteria with a description of the study to obtain written informed consent and administered the University of California, San Diego Brief Assessment of Capacity^25^ to establish their capacity to consent. We enrolled 60 young FEP patients at baseline for the PRS-FEP study. We determined the sample size based on the guidelines for a pilot RCT.^26^

### Measurement

#### Needs

Our study utilised a research version of Camberwell Assessment of Need (CAN-R), which measures the health and social needs of people with severe and enduring mental disorders on a 22-domain scale, its validity having been validated in African settings, including SA.^19^ The list of 22 domains is noted. The first section explores if the need exists using a three-point Likert scale: 0 = no need, 1 = there is a need (met need) and 2 = unmet need (a serious problem because of lack or despite continuing intervention). The second section explores formal and informal help received and formal help needed, assessed using the three-point Likert scale for 0 = no support, 1 = moderate support and 2 = high support. The third section assesses each type of help received, determining whether the right help was received (0 = no, 1 = yes) and if the patient is satisfied with the type of help received (0 = not satisfied, 1 = satisfied). In this study, ‘informal’ sources comprised family, relatives and friends, while ‘formal’ sources included local community services, such as local health and social services and faith-based organisations. For example, in domain number 7 (psychotic symptoms), the first question assessed if the person had any psychotic symptoms, rated 0 = no problem (no positive symptoms, not at risk from symptoms), 1 = no/moderate problem because of help given (symptoms helped by medication or other help), 2 = serious problem (currently has symptoms or at risk) and 3 = not known. These are followed by questions on how much help the person received from friends and local services: 0 = none, 1 = low help (some sympathy and support), 2 = moderate help (care involved in helping with coping strategies or medication compliance), 3 = high help (constant supervision of medication and help with coping) and the help needed from the local services, 0 = none, 1 = low help (less support group), 2 = moderate help (medication reviewed more than 3 monthly), 3 = high help (crisis care at home). With the right type of help received, 0 = no, 1 = yes, and the level of satisfaction, 0 = not satisfied, 1 = satisfied. The benefits and money domains assess distinct aspects of financial needs. The benefits domain evaluates whether individuals receive the financial assistance they are entitled to, while the money domain assesses their ability to pay bills and manage their budget.

#### Food and water security

Household food insecurity was measured using the Household Food Insecurity Access Scale (HFIAS) version 3, which is widely used and validated in developing countries, including SA.^27^ Household Food Insecurity Access Scale consists of a culturally invariant set of nine questions covering three domains related to household food insecurity: (1) anxiety/uncertainty about food supply, (2) altering the quality of the diet and (3) insufficient food intake. The response to each question is based on four choices (no = 0, rarely = 1, sometimes = 2, often = 3). The composite scores are classified into food secure, mildly food-insecure, moderate food-insecure and severe food-insecure using a guideline.^28^ Household water security was measured using the Household Water Insecurity Experiences (HWISE) Scale, which asks respondents to reflect on their experiences of water availability, accessibility, use, acceptability and reliability throughout the previous 4 weeks.^29^ It consists of 12 questions with a score range from 0 to 36, a score of ≥ 12 indicating water insecurity.^29^ The questionnaire was also used to collect the study participants’ socio-demographic information and other clinical characteristics.

### Data collection

A trained research assistant holding an honours degree in psychology and fluent in English and the local KwaZulu-Natal (KZN) language (isiZulu) conducted interviews with participants from July 2023 to December 2023. These took place at the hospital for those still admitted and at the participants’ households for those discharged. The data were captured using REDCap.

### Data analysis

STATA 18 was used, and two types of analyses were conducted, the first being descriptive analyses (e.g. frequency, mean and median) to describe the socio-demographic/clinical profiles of study participants. Descriptive methods were also applied to analyse CAN-R data to describe the level of needs (e.g. no need, met/partially met needs, severe unmet needs), level of help needed from local services (e.g. none, low help, moderate help and high help needed) and type of help received and satisfaction with help. The number of domains for which support (help) was not provided from formal and informal sources, and the proportion of study participants who reported the right help and satisfactory support, was calculated. The second analysis involves a bivariate method investigating factors associated with severe unmet needs. Although this is a pilot study and not powered by any bivariate method, we assessed differences in the total number of severe unmet needs among different socio-demographic and clinical factors using a student’s *t*-test (or ANOVA [analyses of variance] if there are more than two categories).

### Ethical considerations

The PRS-FEP study was approved by the Biomedical Research Ethics Committee (BREC, 00004117/2022) and the KZN Department of Health (KZ_2002209_033) and registered with the South African National Clinical Trial Registry (#DOH- 27- 092022- 5894). Informed consent was obtained from all study participants before data collection.

## Results

### Socio-demographic and other characteristics (*n* = 60)

The sample consisted of 60 participants, mainly male 47 (78.33%) and black African 58 (96.67%), with a median age of 23 years (interquartile range [IQR] = 20–25.5). More than half had completed secondary school or a bachelor’s degree (*n* = 34, 56.67%), and all participants were single, with the majority (*n* = 50, 83.33%) having no children. Approximately, half (*n* = 28, 46.67%) resided in rural areas, and nearly half (*n* = 28, 46.67%) received no personal grants. Most participants (*n* = 58, 96.67%) lived with their families, with an average size of 5.65 ± (2.96), and 36 (60%) reported an average household income below the food poverty line. Half (*n* = 30, 50%) of the participants were diagnosed with schizophrenia, a similar proportion had a history of smoking tobacco, and three-fourths (*n* = 44, 73.33%) self-reported as human immunodeficiency virus (HIV)-negative (Table 1 and Table 2).

**TABLE 1:** Socio-demographic of the study participants (*N* = 60).

Variable	Groups	*n*	%	Median	IQR	Mean	s.d.
Gender	Male	47	78.3	-	-	-	-
	Female	13	21.7	-	-	-	-
Age		-	-	23	20–25.5	-	-
Education	Less than secondary school	26	43.3	-	-	-	-
	Secondary school or bachelor’s completed	34	56.7	-	-	-	-
Race/ethnicity	Black people	58	96.7	-	-	-	-
	White people	2	3.3	-	-	-	-
Marital status	Married	0	0.0	-	-	-	-
	Single	60	100.0	-	-	-	-
Number of children	With no child	50	83.3	-	-	-	-
	With a child	10	16.7	-	-	-	-
Residence	Urban or township	24	40.0	-	-	-	-
	Rural	28	46.7	-	-	-	-
	Informal settlements	8	13.3	-	-	-	-
Individual grant status	Child support, grants and social relief of distress grants	32	53.3	-	-	-	-
	No grant	28	46.7	-	-	-	-
Household average income	Lower food poverty line	36	60.0	-	-	-	-
	Food poverty line	6	10.0	-	-	-	-
	Lower bound poverty line	7	11.7	-	-	-	-
	Upper or above the upper bound poverty line	11	18.3	-	-	-	-
Source of income of the household	Formal employment	20	33.3	-	-	-	-
	Informal employment	21	35.0	-	-	-	-
	Social grants	19	31.7	-	-	-	-
Living status	Living with the family	58	96.7	-	-	-	-
	Living alone	2	3.3	-	-	-	-
Number of individuals living in the household	-	-	-	-	-	5.6	3.0

s.d., standard deviation; IQR, interquartile range.

**TABLE 2:** Clinical and other characteristics of the study participants (*N* = 60).

Variable	Groups	*n*	%
Psychosis diagnosis	Schizophrenia	30	50.0
	Bipolar	9	15.0
	Other psychotic disorders	21	35.0
HIV status (Self-reported)	Positive	6	10.0
	Negative	44	73.3
	Unknown	10	16.7
Substance use (current) multiple response	Tobacco	11	18.3
	Alcohol	6	10.0
	Cannabis	9	15.0
	other	1	1.7
Substance use (lifetime) multiple response	Tobacco	31	51.7
	Alcohol	5	8.3
	Cannabis	23	38.3
	other	1	1.7
Food insecurity	Food secure	2	3.3
	Mildly food-insecure	4	6.7
	Moderate food-insecure	11	18.3
	Severe food insecurity	43	71.7
Water insecurity	Water security	38	63.3
	Water insecurity	22	36.7

HIV, human immunodeficiency virus.

### Need assessment using Camberwell Assessment of Need

Participants did not identify needs in five domains: looking after home, drugs, sexual expression, telephone access and transport. The more frequent met needs were associated with psychotic symptoms, information on the condition and treatment, and food, while the benefits domain reported the highest frequency of (severe) unmet needs (Table 3). Regarding their needs, most participants indicated receiving assistance from the family and relatives (informal source). The mean number of domains for which support (help) was not provided from informal and formal sources was 2.63 (standard error [s.e.] = 0.25) and 4.44 (s.e. = 0.29), with the latter being significantly higher (M_diff_= −1.78, *t* = −8.89, *p* = 0.00). Most participants indicated considerable help being needed from their local services, with many reporting that they were not receiving the right type of help and were not satisfied with the help received (Table 5 and Table 6).

**TABLE 3:** Assessment of the level of needs for 22 items in Camberwell Assessment of Need (*N* = 60).

Domain	No need		Met or partially met		Severe unmet	
	*n*	%	*n*	%	*n*	%
Accommodation	57	95.0	2	3.3	1	1.7
Food	38	63.3	20	33.3	2	3.3
Looking after home	60	100.0	0	0.0	0	0.0
Self-care	58	96.7	1	1.7	1	1.7
Daytime activities	57	95.0	0	0.0	3	5.0
Physical health	50	83.3	8	13.3	2	3.3
Psychotic symptoms	6	10.0	53	88.3	1	1.7
Information on condition and treatment	24	40.0	31	51.7	5	8.3
Psychological distress	28	46.7	28	46.7	4	6.7
Safety to self	48	80.0	9	15.0	3	5.0
Safety to others	58	96.7	2	3.3	0	0.0
Alcohol	58	96.7	2	0.3	0	0.0
Drugs	60	100.0	0	0.0	0	0.0
Company	51	85.0	1	1.7	8	13.3
Intimate relationship	59	98.3	1	1.7	0	0.0
Sexual expression	60	100.0	0	0.0	0	0.0
Childcare	50	83.3	10	16.7	0	0.0
Basic education	49	81.7	6	10.0	5	8.3
Telephone	60	100.0	0	0.0	0	0.0
Transport	60	100.0	0	0.0	0	0.0
Money	49	81.7	2	3.3	9	15.0
Benefits	9	15.0	19	31.7	32	53.3

Note: More than half (*n* = 32/60) of the study participants reported a severe unmet need from the benefit domain.

**TABLE 4:** Factors associated with severe unmet need among study participants (*N* = 60).

Variable	Groups	Observations	Mean	s.d.	Student *t*-test	*f-test*	*P*-value
Gender	Female	13	1.15	0.45	−0.41	-	0.650
	Male	47	1.36	0.23	-	-	-
Education	Less than secondary school	26	1.84	0.36	2.24	-	0.003
	Secondary school and above	34	0.91	0.22	-	-	-
HIV status (self-reported)	Positive	6	1.33	0.52	-	0.31	0.730
	Negative	44	1.27	1.69	-	-	-
	Unknown	10	1.50	1.84	-	-	-
Household average income	Lower food poverty line	36	1.38	1.77	-	0.31	0.860
	Food poverty line	6	1.50	2.25	-	-	-
	Lower bound poverty line	7	1.00	1.15	-	-	-
	Upper or above upper bound poverty line	11	1.18	0.95	-	-	-
Grant status	Child support and SRD grants	32	1.34	1.49	0.35	-	0.720
	No grant	28	1.28	1.78	-	-	-
Number of children	With no child	50	1.30	1.60	1.25	-	0.210
	With a child	10	1.40	1.77	-	-	-
Residence	Urban and township	24	0.95	1.30		1.91	0.150
	Rural	28	1.42	1.81	-	-	-
	Informal settlements	8	2.00	1.62	-	-	-
Diagnosis	Schizophrenia	30	1.40	1.77	-	0.56	0.640
	Bipolar	9	1.00	0.86	-	-	-
	Other psychosis	21	1.33	1.77	-	-	-
Water security	Water security	38	1.09	1.33	0.71	-	-
	Water insecurity	22	1.78	2.07	-	-	-
Food security	Food secure	2	0.50	0.71	-	1.60	0.200
	Mild food-insecure	4	0.50	0.58	-	-	-
	Moderate food-insecure	11	0.64	0.81	-	-	-
	Severe food insecurity	45	1.60	1.79	-	-	-

HIV, human immunodeficiency virus; SRD, social relief of distress; s.d., standard deviation.

**TABLE 5:** Level of help needed from local services from each domain.

Domain	None		Low help		Moderate help		High help		Total need	
	*n*	%	*n*	%	*n*	%	*n*	%	*n*	%
Accommodation	0	0.0	1	33.3	2	66.7	0	0.0	3	100.0
Food	0	0.0	9	40.9	6	27.3	7	31.9	22	100.0
Self-care	0	0.0	1	50.0	0	0.0	1	50.0	2	100.0
Daytime activities	0	0.0	2	66.7	1	33.3	0	0.0	3	100.0
Physical health	1	10.0	0	0.0	9	90.0	0	0.0	10	100.0
Psychotic symptoms	19	35.2	26	48.1	9	16.7	0	0.0	54	100.0
Information on condition and treatment	1	2.8	34	94.4	1	2.8	0	0.0	36	100.0
Psychological distress	3	9.4	19	59.4	9	28.1	1	31.1	32	100.0
Safety to self	2	16.7	6	60.0	4	33.3	0	0.0	12	100.0
Safety to others	0	0.0.	1	50.0	0	0.0	1	50.0	2	100.0
Alcohol	2	100.0	0	0.0	0	0.0	0	0.0	2	100.0
Company	2	22.2	7	77.8	0	0.0	0	0.0	9	100.0
Intimate relationship	1	100.0	0	0.0	0	0.0	0	0.0	1	100.0
Childcare	2	20.0	1	10.0	7	70.0	0	0.0	10	100.0
Basic education	3	27.3	7	63.7	1	9.1	0	0.0	11	100.0
Money	1	9.1	4	36.4	6	54.5	0	0.0	11	100.0
Benefits	3	5.9	14	27.5	6	11.7	28	54.9	51	100.0

Note: More than half (*n* = 28/51) of the study participants reported a need for high help from total needs in the benefit domain.

**TABLE 6:** Help received and level of satisfaction in each domain with a need.

Domain	Right help				Satisfied				Total need	
	Yes		No		Yes		No			
	*n*	%	*n*	%	*n*	%	*n*	%	*n*	%
Accommodation	0	0.0	3	100.0	0	0.0	3	100.0	3	100.0
Food	0	0.0	22	100.0	0	0.0	22	100.0	22	100.0
Self-care	0	0.0	2	100.0	0	0.0	2	100.0	2	100.0
Daytime activities	0	0.0	3	100.0	0	0.0	3	100.0	3	100.0
Physical health	2	20.0	8	80.0	2	20.0	8	80.0	10	100.0
Psychotic symptoms	43	79.6	11	20.4	37	68.5	17	31.5	54	100.0
Information on condition and treatment	2	5.6	34	94.4	2	5.6	34	94.4	36	100.0
Psychological	1	3.1	31	96.9	0	0.0	32	100.0	32	100.0
Safety to self	1	8.3	11	91.7	0	0.0	12	100.0	12	100.0
Safety to others	0	0.0	2	100.0	0	0.0	2	100.0	2	100.0
Alcohol	1	50.0	1	50.0	1	50.0	1	50.0	2	100.0
Company	1	11.1	8	88.9	1	11.0	8	88.9	9	100.0
Intimate relationship	0	0.0	1	100.0	0	0.0	1	100.0	1	100.0
Childcare	2	20.0	8	80.0	2	20.0	8	80.0	10	100.0
Basic education	0	0.0	11	100.0	0	0.0	11	100.0	11	100.0
Money	1	9.1	10	90.9	1	9.1	10	90.9	11	100.0
Benefits	1	1.9	50	98.1	0	0.0	51	100.0	51	100.0

Note: Most of the study participants received appropriate help for psychotic symptoms, with over half of the participants expressing satisfaction with the assistance they received. Also, in most domains, participants reported not receiving the appropriate help and were dissatisfied with the assistance.

### Food and water insecurity

Among the 60 participants, two (3.3%) were food-secure, four (6.7%) were mildly food-insecure, 11 (18.3%) were moderately food-insecure and 43 (71.7%) were severely food-insecure, while 22 (36.7%) reported being water-insecure.

### Factors associated with total severe unmet needs

The only factor that shows a significant mean difference is education; the mean of the severe unmet need is twice among participants with less than secondary education compared to the participants with secondary or bachelor’s education; other factors were not statistically significant (Table 4).

## Discussion

This study assessed the level of need among 60 young adults following FEP. Firstly, our research report reveals a substantial unmet need because of difficulties in accessing government benefits to which individuals are entitled, coupled with limited information and communication regarding their medical conditions. Secondly, although access to mental health services remains a challenge in SA, most participants reported having their needs either fully or partially met in terms of managing psychotic symptoms. Lastly, a significant portion of youth expressed dissatisfaction with the type of support they received.

Most participants rated the lack of government benefits as a severe unmet need, expressing dissatisfaction with the assistance provided by family and relatives, with only one acknowledging receiving support from local services. This aligns with our expectations as all study participants are unemployed and rely on government benefits. A few studies conducted in Chile and Canada have reported similar findings despite differences in population profiles and national income levels,^30,31^ possibly as psychosis often disrupts plans and goals, mainly when onset occurs at a young age, impacting skills development. In this study, about half of the participants reported receiving the social relief of distress (SRD) grant of R350 per month, even though all participants were unemployed and therefore qualified for this support. This discrepancy highlights potential barriers to accessing social protection, such as administrative challenges, a lack of awareness or systemic inefficiencies. The government needs to strengthen and streamline the mechanisms for assessing eligibility and distributing grants in a timely and efficient manner to ensure that individuals with disabling mental health conditions receive the financial support to which they are entitled.

Also, most participants reported a need for information about their condition and available treatments. This indicates a communication gap between patients and their healthcare professionals, consistent with findings from studies conducted in Spain and the United Kingdom.^32,33^ Despite differences in population characteristics and the availability of mental health resources, similar gaps in communication and patient education were observed. This suggests that providing clear, accessible information remains a fundamental requirement of mental healthcare across diverse contexts.

Nearly half of the study sample had been hospitalised during this baseline assessment, with management efforts focused more on their acute challenges, leaving other need domains unaddressed in the initial stages of treatment. Improving communication in healthcare is crucial for ensuring that patients fully understand their conditions, treatment options and potential outcomes. Effective communication can lead to better-informed decisions, increased adherence to treatment and improved patient outcomes. It is essential to create a collaborative and supportive healthcare environment where patients feel empowered and informed about their health and treatment options. Healthcare workers require training on the importance of delivering comprehensive care, particularly in mental health, with an emphasis on using psychoeducation to inform and empower patients about their conditions. In addition, improving the quality of care should focus on strategies implemented within the existing human resource capacity, without reliance on external interventions, by optimising the use of available resources effectively.

Although mental health services in South Africa face significant challenges related to access because of resource constraints and pervasive stigma,^34^ it was surprising to find in this study that the majority of participants indicated having met or partially met needs in the domain of psychotic symptoms. This outcome is likely because all participants were recruited from an ongoing study project, ensuring that they had some level of engagement with mental health services. This engagement may have facilitated better access to necessary treatments and support. Also, all participants reported receiving antipsychotic medication for treatment, this being similar to the previous literature.^30^ Both studies included participants with FEP, and this may be because of their early stages of the disease, the need of being associated with significant changes in their health conditions.

In this study, none of the participants reported receiving assistance from local services or referring to community health services for psychotic symptoms. Resource constraints in community mental health services and stigma and discrimination associated with mental health patients may contribute to the patient’s failure to seek help from local services for psychotic symptoms, especially at the early stages of the illness.^35^ Also, the structure of the healthcare system may influence the limited support from local services. During the initial months of treatment, patients are typically monitored within hospital settings until their condition is deemed stable by the attending psychiatrist. Transition to community care is initiated through a referral made by the treating hospital psychiatrist. However, the lack of data on care pathways introduces uncertainty, as some participants may have been referred from primary healthcare services. In practice, individuals with FEP are often directed to higher-level facilities for comprehensive diagnosis and evaluation. This underscores the need for policymakers and mental health authorities to re-evaluate existing support structures, particularly at the primary healthcare level, to strengthen accessibility and ensure that mental health services are comprehensive, inclusive and responsive to the needs of all community members.

Our assessment found that none of the participants expressed a need for transport because of limitations in how the tool addresses transportation needs. The CAN tool primarily evaluates access to public transportation. Still, it does not explicitly consider financial barriers, such as participants’ ability to afford fares, which could prevent consistent access even if public transport is technically available. This narrow scope may overlook significant barriers, particularly for those with mental health conditions who need reliable, affordable transportation to access essential services. Further assessment, which includes questions about affordability and transportation reliability, could provide a more comprehensive view of participants’ needs, enabling mental health professionals to deliver better-targeted support.

Climate change has negatively affected physical and mental health directly and indirectly,^36^ the prolonged drought because of climate change results in food and water insecurity.^37^ Although a small proportion of participants indicated unmet needs in the food domain assessed by CAN-R, many fell under severe food insecurity after the HFIAS assessment, similar to a recent systematic review among mental health patients.^38^ These patients are subjected to considerable life challenges, especially in cognitive functioning and employment, which increase the risk of financial difficulties and, therefore, access to food.^39^ Studies have shown that a lack of food is associated with poor medication adherence in HIV patients. A few studies performed to assess the reasons for poor medication adherence in mental health patients report an association with a lack of food.^40^

In addition, individuals who experience water scarcity may focus on meeting their immediate needs, affecting medication adherence.^41^ Medication adherence has also been reported to be the critical determinant for single-episode psychosis among patients with mental health problems.^42^ Early intervention services that include the provision of or access to food after FEP will help affected persons to meet their basic needs and adhere to their treatment.

In this study, we anticipate identifying a higher number of reported needs from participants because of the early stage of their psychosis. Our expectation aligns with our findings, indicating that the number of unmet needs in this sample is higher than in studies that examined needs in the severe mental health population,^4,19^ possibly because of the early stage of their illness. The additional needs may be associated with the initial stage of the disease, often accompanied by worries and fear of management. These data underscore the importance of early intervention and comprehensive support for those newly diagnosed with psychosis.

Lastly, most participants expressed dissatisfaction with the support they received, facing substantial unmet needs in accessing government benefits. All were unemployed, and the pressures of meeting essential survival needs often took priority, limiting their ability to continue with necessary care. Despite the availability of disability grants in SA, no participants received these benefits, highlighting the need for enhanced social security measures. Although cash transfer programmes have proven to be effective in addressing basic needs, there is limited evidence of their feasibility and acceptability for individuals with FEP. Therefore, a feasibility study is needed to evaluate whether UCTs can effectively support health needs and continuity of care for individuals with FEP.

### Study’s limitation

This study was conducted at government hospitals; nonetheless, a large number of people in South Africa get specialised mental healthcare from public hospitals. Future studies should consider including a community sample and a private hospital, as they may have different needs.

### Contribution

This is the first study that assesses the needs of young adults with FEP in KwaZulu-Natal province who access care through the public sector. The needs identified in this study can be used to design treatment plans for patients with FEP to help them engage in care.

## Conclusion

A serious unmet need in the benefits domain indicates the need for financial assistance to help young FEP patients engage in care in the early stage of the disease to prevent its progression into the chronic stage. Poor coordination between hospital and community mental health services exacerbates the lack of support at the local level. There is a need to strengthen mental health support services at the community level to improve detection and treatment adherence through collaboration with specialised hospitals.
